# Age-Dependent Changes in Nuclear Mechanotransduction as a Driver of Sarcopenia

**DOI:** 10.1093/geroni/igaa057.424

**Published:** 2020-12-16

**Authors:** Shama Iyer, Christopher Ward, Joseph Stains, Alice Ryan, Eric Folker, Richard Lovering

**Affiliations:** 1 University of Maryland School of Medicine, Baltimore, Maryland, United States; 2 Baltimore VA Medical Center, Baltimore, Maryland, United States; 3 Boston College, Chestnut Hill, Massachusetts, United States

## Abstract

Informed by evidence that dysregulated nuclear dynamics and nuclear transport may contribute to atrophy in diseased skeletal muscle, the purpose of this study was to assess nuclear deformability, permeability, transport, and mechano-signaling outputs (YAP/TAZ, a marker of mechano-responsiveness, and their downstream genes) in aging skeletal muscle. We hypothesized that aging muscle would show changes in: proteins within LINC (linker of the nucleus to the cytoskeleton) complex, lamina and nuclear pore complex (NPC), and mechano-signaling outputs, with consequent decreased nuclear deformability and increased permeability. We further expected an increase in nuclear strain would increase nuclear YAP/TAZ and downstream indicators of YAP activity (Ankrd1, Cyr61). We used young, adult and aged C57BL6 mice (~4, 14, and 26 months, respectively). Nuclei were less deformable to passive mechanical stretch ex-vivo in adult muscle fibers compared to young muscle fibers. LINC protein gene expression, YAP/TAZ protein, and expression of their downstream genes were significantly increased in adult muscles compared to young muscles. YAP/TAZ protein and their downstream genes were further increased in aged muscles, indicating hyperactivation of YAP/TAZ in aging muscle. Changes with aging in the lamina and NPC included a loss of lamin β1, Nup107 and POM 121, which could underlie the increased nuclear permeability we found in nuclei of aged muscle. In summary, these data highlight a possible role for LINC, lamina and NPC in changes of aging-related nuclear dynamics and mechano-sensing, and may represent therapeutic targets for sarcopenia. Future studies will examine how altering these components affects muscle function during aging.

